# A New Species of *Curcuma* subgen. *Ecomatae* (Zingiberaceae) from Southwestern Thailand: Morphological Delimitation and Phenetic Evidence †

**DOI:** 10.3390/plants15193072

**Published:** 2026-10-08

**Authors:** Thawatphong Boonma, Surapon Saensouk, Piyaporn Saensouk, Pathomthat Srisuk, Suriya Phimpha, Prasert Ruannakarn, Kamonwan Koompoot, Win Paing Oo

**Affiliations:** 1 Diversity of Family Zingiberaceae and Vascular Plant for Its Applications Research Unit, Walai Rukhavej Botanical Research Institute, Mahasarakham University, Maha Sarakham 44150, Thailand; boonma.thawat@gmail.com (T.B.); kamonwan.k@kkumail.com (K.K.); 2 Diversity of Family Zingiberaceae and Vascular Plant for Its Applications Research Unit, Department of Biology, Faculty of Science, Mahasarakham University, Maha Sarakham 44150, Thailand; 3 Department of Pharmaceutical Technology, Faculty of Pharmaceutical Sciences, Khon Kaen University, Khon Kaen 40002, Thailand; spatho@kku.ac.th; 4 Faculty of Humanities and Social Sciences, Mahasarakham University, Maha Sarakham 44150, Thailand; suriya.pi@msu.ac.th; 5 Department of Educational Research and Development, Faculty of Education, Mahasarakham University, Maha Sarakham 44000, Thailand; prasert.rua@msu.ac.th; 6 College of Sustainability and Tourism, Ritsumeikan Asia Pacific University, Beppu 874-8577, Oita, Japan; winpaingoo2019@gmail.com

**Keywords:** Gower distance, morphological differentiation, morphological variation, numerical taxonomy, phenetic analysis, Tenasserim range

## Abstract

*Curcuma* subgen. *Ecomatae* comprises several morphologically similar taxa whose delimitation requires careful evaluation of character combinations and intraspecific variation. Botanical surveys across Southwestern Thailand revealed six geographically separated populations of an unidentified *Curcuma* in Uthai Thani, Kanchanaburi, Ratchaburi, Phetchaburi, and Prachuap Khiri Khan provinces. Taxonomic status was evaluated through detailed comparative morphology and complementary individual-level phenetic analyses of 42 morphological characters using Gower distances, UPGMA clustering, and principal coordinates analysis (PCoA). The analyses comprised a population-level assessment of 120 individuals from six populations and an interspecific comparison of 200 individuals, including the same 120 individuals of the putative new taxon and 20 individuals each of four morphologically allied species. The populations are recognized here as a distinct species, *Curcuma tenasserimensis* Boonma, Saensouk & P.Saensouk, sp. nov., based on a consistent combination of vegetative, inflorescence, floral, and reproductive characters that distinguishes them from the morphologically allied species examined. The population-level analysis revealed morphological variation among the six geographically separated populations while retaining their overall morphological cohesion. In the interspecific analysis, individuals of *C. tenasserimensis* formed an overall morphologically cohesive assemblage that was differentiated from the four allied species examined. Together, comparative morphology and individual-level phenetic analyses are consistent with the recognition of *C. tenasserimensis* as a morphologically distinct species and further document the still-emerging diversity of subgen. *Ecomatae* in Southwestern Thailand.

## 1. Introduction

Species recognition in *Curcuma* subgen. *Ecomatae* Škorničk. & Šída f. [1] can be challenging because several taxa share broadly similar vegetative habits and floral features, while some quantitative characters vary among populations [2,3]. Nevertheless, continued taxonomic studies in mainland Southeast Asia have demonstrated that stable combinations of vegetative, inflorescence, and floral characters can distinguish morphologically coherent taxa [4,5,6,7]. Thailand harbors substantial diversity within subgen. *Ecomatae*, and recent discoveries indicate that its species diversity and geographical variation remain incompletely documented, particularly in areas that have received comparatively limited botanical exploration [4,5,8,9].

Southwestern Thailand represents a floristically distinctive part of the country, characterized by a heterogeneous landscape of low hills, forest margins, valleys, and evergreen forest habitats. The region lies within the broader geographical context of the Tenasserim landscape of western Thailand and adjacent Myanmar, where complex topography contributes to considerable habitat heterogeneity [10,11,12]. The forest habitats within this landscape support diverse plant assemblages, yet the diversity and distribution of several herbaceous groups, including Zingiberaceae, remain insufficiently documented [2,5]. Continued field exploration is therefore important for documenting plant diversity and for determining whether morphologically distinctive populations represent previously unrecognized taxa or geographical variation within already known species.

Species delimitation based exclusively on a small number of diagnostic characters can be problematic when morphological variation overlaps among taxa. Detailed comparative morphology remains fundamental to plant taxonomy, while multivariate phenetic approaches provide a complementary means of evaluating overall morphological similarity using multiple qualitative and quantitative characters simultaneously [13,14]. Similarity analysis, clustering, and ordination can therefore provide descriptive summaries of overall morphological similarity and differentiation among putative taxa, recognized species, and geographically separated populations. Such analyses evaluate phenetic similarity and morphological differentiation and are not intended to infer phylogenetic relationships.

During botanical investigations of Zingiberaceae in the Southwestern floristic region of Thailand, populations of an unusual *Curcuma* belonging to subgen. *Ecomatae* were collected from Uthai Thani, Kanchanaburi, Ratchaburi, Phetchaburi, and Prachuap Khiri Khan provinces. These populations occur mainly in mixed evergreen forest and along forest margins on low hills at elevations of approximately 50–320 m a.s.l. Detailed examination across the known geographical range revealed a consistent combination of vegetative, inflorescence, and floral characters that could not be attributed to any currently recognized species. Although some quantitative characters vary among populations, the principal diagnostic features remain consistent across geographically separated localities. Accurate taxonomic recognition is also fundamental to documenting locally utilized plant resources and establishing an appropriate basis for their conservation and sustainable management.

The present study aims to clarify the taxonomic identity of these populations through detailed comparative morphology complemented by quantitative phenetic analyses. The taxonomic treatment includes a description and illustration of *Curcuma tenasserimensis* Boonma, Saensouk & P.Saensouk, sp. nov., together with information on its distribution, ecology, phenology, preliminary conservation status, and morphological differentiation from related members of subgen. *Ecomatae*. Phenetic analyses based on Gower distances, UPGMA clustering, and principal coordinates analysis (PCoA) were conducted at two complementary levels using individual plants as operational taxonomic units (OTUs): an intraspecific analysis of 120 individuals from six geographically separated populations of *C. tenasserimensis* to evaluate population-level morphological variation, and an interspecific analysis of 200 individuals comprising the same 120 individuals of *C. tenasserimensis* together with 20 individuals each of four morphologically allied species. These analyses are used descriptively to quantify patterns of morphological variation, cohesion, and differentiation, thereby providing a broader morphological framework for the taxonomic assessment of the new species.

## 2. Results

### 2.1. Taxonomic Treatment

***Curcuma tenasserimensis* Boonma, Saensouk & P.Saensouk, sp. nov.**

**Figure 1:**
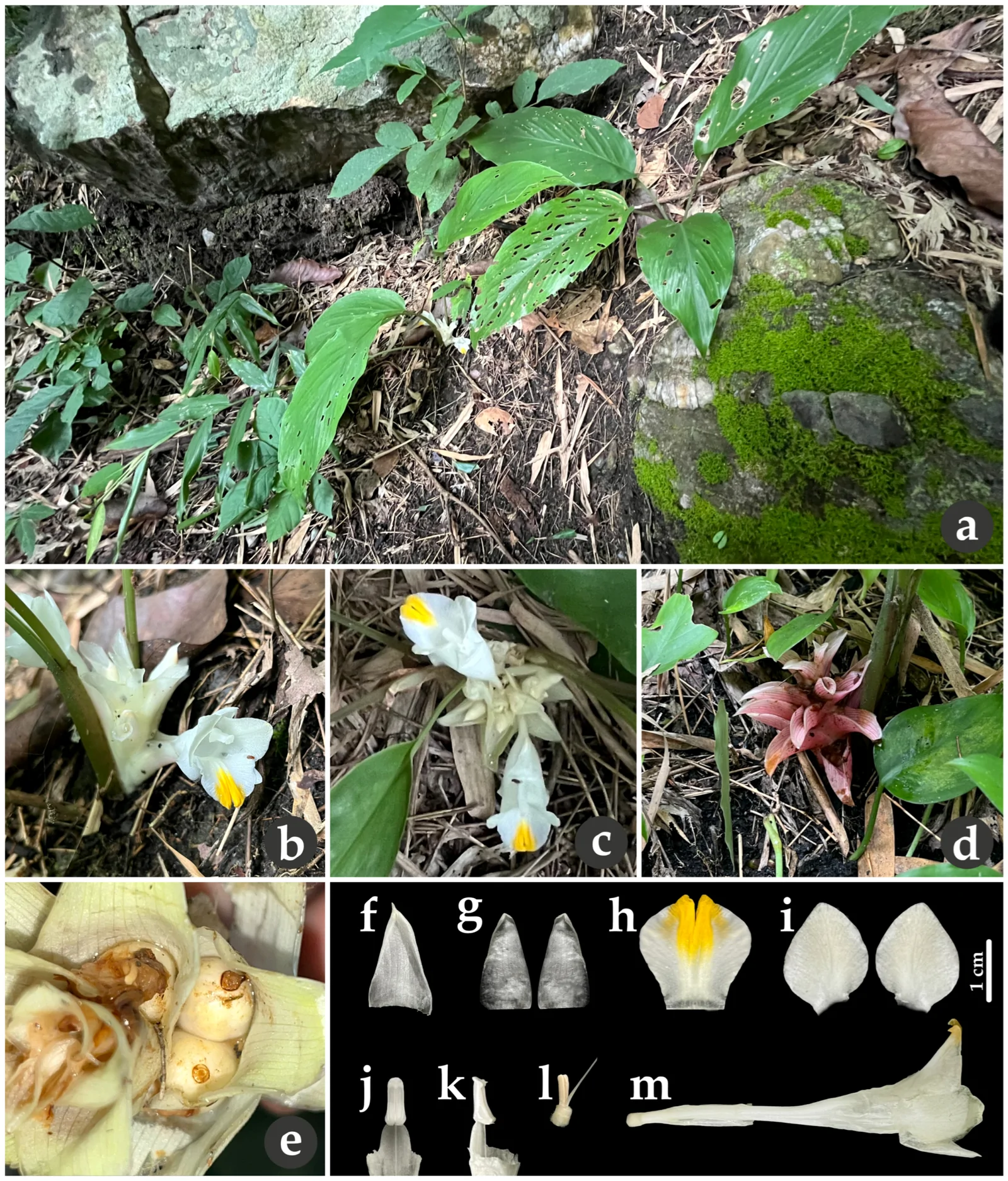
*Curcuma tenasserimensis* Boonma, Saensouk & P.Saensouk, sp. nov.: (**a**) Habits in natural habitat. (**b**) Lateral view of inflorescence and flower. (**c**) Top view of inflorescence and flower. (**d**) Pinkish fertile bracts. (**e**) Fruits in inflorescence. (**f**) Dorsal corolla lobe. (**g**) Lateral corolla lobes. (**h**) Labellum. (**i**) Lateral staminodes. (**j**) Front view of stamen. (**k**) Lateral view of stamen. (**l**) Epigynous glands and ovary. (**m**) Flower with calyx and ovary. Photographs by Thawatphong Boonma.

**Figure 2:**
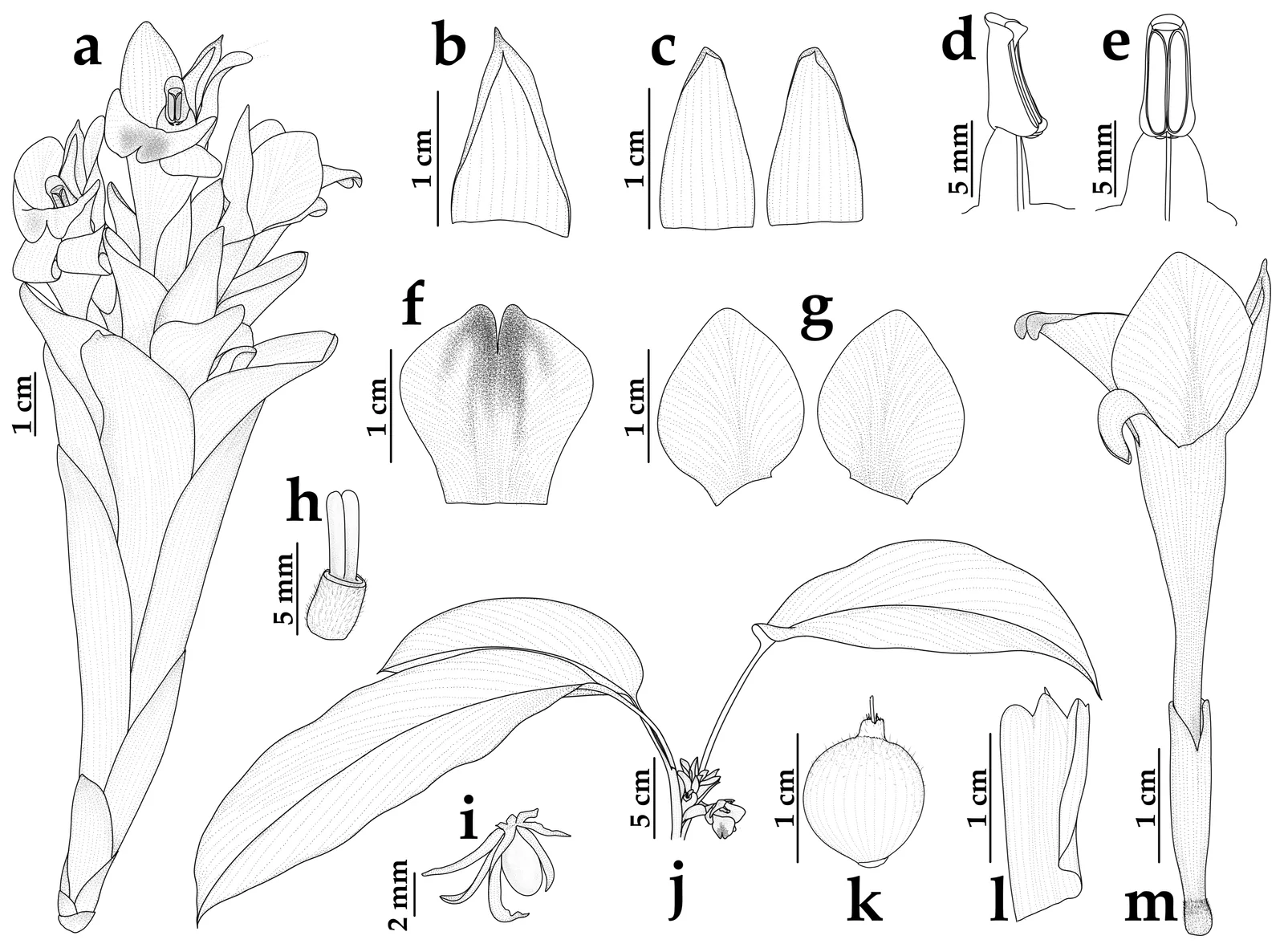
*Curcuma tenasserimensis* Boonma, Saensouk & P.Saensouk, sp. nov.: (**a**) Inflorescence with flowers and sterile bracts. (**b**) Dorsal corolla lobe. (**c**) Lateral corolla lobes. (**d**) Lateral view of stamen. (**e**) Front view of stamen. (**f**) Labellum. (**g**) Lateral staminodes. (**h**) Epigynous glands and ovary. (**i**) Seed with arils. (**j**) Habit. (**k**) Fruit. (**l**) Dissected calyx. (**m**) Lateral view of flower. Photographs by Thawatphong Boonma.

**Type:** THAILAND. Ratchaburi Province, Suan Phueng District, 305 m a.s.l., 19 October 2024, T.Boonma RB2401 (holotype VMSU! [VMSU00000016]; isotype BKF!) (Figure 1 and Figure 2).

**Diagnosis.***Curcuma tenasserimensis* is morphologically most similar overall to *C. pierreana*, but differs in having a longer ligule, 6–8 mm long (vs. c. 2 mm); the peduncle mostly enclosed by the leaf sheaths and the basal portion of the thyrse further enclosed by sterile basal bracts (vs. peduncle and basal portion of the thyrse exserted); fertile bracts ovate to lanceolate (vs. broadly ovate); lateral staminodes and labellum lacking reddish-purple coloration distally (vs. usually reddish purple distally); a labellum with a yellow distal portion gradually fading proximally (vs. a bright yellow median band, usually reddish purple apically); and a longer filament, c. 5 mm long (vs. 2–3 mm long). It is also morphologically similar to *C. eburnea* in having prominently plicate laminae, inflorescences without coma bracts, pale-coloured fertile bracts and flowers, and spurred anthers with a thick, rounded crest. It differs from *C. eburnea* by having the peduncle mostly enclosed by the leaf sheaths and the basal portion of the thyrse additionally enclosed by sterile basal bracts (vs. peduncle and basal portion of the thyrse exserted from the leaf sheaths); 12–18 fertile bracts (vs. 30–40), connate in the lower 1/5–1/3 (vs. lower 1/3–1/2); bracteoles present (vs. absent); cincinni with 3–4 flowers (vs. 1–3); a longer, puberulent calyx, 15–20 mm long (vs. 8–10 mm long and glabrous); an obovate, shortly hairy labellum with a yellow distal portion gradually fading towards approximately half of the median band (vs. an obscurely trilobed, glabrous labellum with a bright yellow median band running through the centre); a longer filament, c. 5 mm (vs. c. 2 mm); longer epigynous glands, c. 5 mm (vs. c. 3 mm); and a subglobose to oblate, puberulous to pilose ovary (vs. cylindrical and glabrous).

**Description.** Perennial herb 30–50 cm tall. Primary rhizome ovoid to subglobose, 1.5–3 × 1–2 cm, ivory internally, fresh rhizome slightly aromatic when cut or damaged; rhizome branches few, slender, 0.5–1 cm in diameter, brown externally, ivory internally. Root tubers ellipsoid to fusiform, c. 2.5–3 × 1.5 cm, light brown externally, pure white internally with a translucent white centre. Leafy shoot with 2–4 leaves when flowering; pseudostem up to 10 cm long, mostly subterranean. Bladeless sheaths green or tinged reddish brown, turning brown at anthesis, apex acute and slightly mucronate, sparsely puberulous. Leaf sheaths green or tinged brownish, puberulent. Ligule to 8 mm long, prominently bilobed, hyaline, greenish white, semitranslucent, turning papery with age, glabrous except for short hairs along margins. Petiole 2–17 cm long, green, sometimes tinged brownish, sparsely puberulent. Lamina ovate, elliptic to oblanceolate, 10–48 × 6–13 cm, prominently plicate, adaxially bright green, occasionally tinged reddish brown along the midrib, glabrous, abaxially pale green, puberulent; midrib green, glabrous; base rounded to cuneate; apex acuminate; margin hyaline, semitranslucent white. Inflorescence terminal. Peduncle 2–6 cm long, very pale green to pale yellowish green or white towards base, densely puberulent, mostly enclosed by sterile bracts and partly embedded within the pseudostem. Thyrse 4–9 cm long, 4–6.1 cm in diameter at the broadest part, with few sterile bracts. Fertile bracts 12–18, ovate to lanceolate, larger towards the base of the inflorescence, 3–4.5 × 1–3 cm, cream, pale green or pale pink, sparsely puberulent to puberulent, margins semitranslucent and hyaline, connate in the lower 1/5–1/3; cincinni with 3–4 flowers. Coma bracts absent. Bracteoles obovate to elliptic, c. 8 × 4 mm, sparsely hairy along the midvein, apex hooded. Flowers 4.5–6.1 cm long, exserted from the fertile bracts. Calyx 15–20 mm long, 3-lobed, with a unilateral incision 3–6 mm long, semitranslucent white, puberulent. Floral tube 3–4.3 cm long, narrowly cylindrical basally and funnel-shaped distally, white to ivory externally, with scattered fine hairs. Dorsal corolla lobe 15–16 × 7–9 mm, triangular-ovate, concave, hooded, ivory to very pale pink, glabrous, apex mucronate; mucro c. 1.5 mm long. Lateral corolla lobes 11–13 × 7–9 mm, triangular-ovate, slightly concave apically, strongly reflexed soon after anthesis, ivory to very pale pink, glabrous. Labellum 15–18 × 15–18 mm, obovate, apex emarginate with an incision to 5 mm deep, ivory to cream with a yellow distal part gradually fading towards approximately half of the median band, with short hairs denser along the margins of the median band. Lateral staminodes 15–20 × 10–16 mm, ovate to broadly elliptic, ivory to cream, occasionally pale yellow distally, sparsely covered with short hairs, apex obtuse. Stamen c. 12 mm long. Filament c. 5 mm long, c. 6 mm broad at base and c. 3 mm broad at apex, white, sparsely puberulent. Anther 8–8.5 mm long, spurred; connective white, sparsely puberulent. Anther spurs c. 1 mm long, filamentous, incurved. Anther crest thick, rounded, c. 1.5 mm long and c. 2.5 mm broad at base, ivory, occasionally tinged very pale yellow distally. Anther thecae c. 6 mm long, white, dehiscing along their entire length; pollen white. Epigynous glands two, c. 5 mm long and c. 1 mm in diameter, cylindrical, ivory, apex blunt. Style white, glabrous. Stigma slightly flattened-capitate, c. 1 × 1.5 mm, ivory; ostiole ciliate, facing forwards. Ovary subglobose to oblate, 2.5–3 × c. 2.5 mm, trilocular, ivory to pale cream, puberulous to pilose. Fruits subglobose to obovoid, 8–10 × 8–10 mm, ivory to pale cream, puberulous. Seeds obovate, brown with white arils.

**Etymology.** The specific epithet “*tenasserimensis*” refers to the Tenasserim Range, reflecting the occurrence of the species in areas associated with this mountain range in Southwestern Thailand.

**Distribution.***Curcuma tenasserimensis* is currently confirmed from six populations in Uthai Thani, Kanchanaburi, Ratchaburi, Phetchaburi, and Prachuap Khiri Khan provinces in the Southwestern floristic region of Thailand.

**Ecology.** The species occurs mainly in mixed evergreen forest and along forest margins on low hills at elevations of approximately 50–320 m a.s.l.

**Phenology.** Flowering from July to October; fruiting from August to November.

**Utilization.** During field surveys in Kanchanaburi and Ratchaburi provinces, flowering shoots of *Curcuma tenasserimensis* were occasionally encountered for sale in local markets, typically tied in small bundles.

**Additional specimens examined.** THAILAND. Southwestern: Uthai Thani, Ban Rai District, elevation 297 m a.s.l., 2 September 2023, *T.Boonma & S.Phimpha UT2391* (VMSU). Kanchanaburi Province, Tha Muang District, elevation 85 m a.s.l., 22 June 2023, *T.Boonma KR2361* (VMSU). Ratchaburi Province, Photharam District, elevation c. 50 m a.s.l, 5 August 2022, *T.Boonma RB2285* (VMSU). Phetchaburi Province, Tha Yang District, elevation c. 120 m a.s.l., 6 August 2022, *T.Boonma PB2281* (VMSU). Prachuap Khiri Khan Province, Hua Hin District, elevation c. 100 m a.s.l., 7 August 2022, *T.Boonma PK2282* (VMSU).

**Preliminary Conservation Assessment.***Curcuma tenasserimensis* is currently known from six geographically separated populations in Uthai Thani, Kanchanaburi, Ratchaburi, Phetchaburi, and Prachuap Khiri Khan provinces, Southwestern Thailand. All six known populations occur outside formally protected areas. GeoCAT analysis estimated an extent of occurrence (EOO) of 4270.253 km^2^ and an area of occupancy (AOO) of 24 km^2^ using a 2 × 2 km grid [15,16,17]. Although the estimated EOO and AOO fall within the geographical thresholds for threatened categories under Criterion B, the available evidence does not satisfy the additional subcriteria required for listing as threatened. All six known populations remain extant, and each was estimated during field surveys to contain more than 200 mature individuals, defined here as plants bearing an inflorescence with at least one open flower. Reproductive activity and recruitment were also evident from the presence of fruiting individuals, seedlings or young plants, and vegetative recruitment through lateral shoots. Repeated field observations have not indicated significant population decline, continuing habitat deterioration, severe fragmentation, or extreme fluctuations. No direct evidence of ongoing population declines attributable to agricultural conversion or harvesting was documented at the known localities. Although flowering shoots have occasionally been observed for sale in local markets, there is currently no evidence that such harvesting has resulted in measurable declines in the known populations. The limited removal of material for voucher preparation and living collections was not considered to impose measurable disturbance on the surveyed populations because only a small proportion of the observed plants was collected. The principal threats remain uncertain, and although periodic drought may affect recruitment in some years, its long-term effects have not been demonstrated. *Curcuma tenasserimensis* is therefore provisionally assessed as Least Concern (LC), while continued monitoring of population trends, habitat condition, and potential future threats is recommended.

The principal morphological differences between *Curcuma tenasserimensis* and the morphologically similar *C. eburnea* and *C. pierreana* are summarized in Table 1.

To facilitate identification, a key to *Curcuma tenasserimensis* and morphologically similar species of *Curcuma* subgen. *Ecomatae* is provided below, based primarily on morphological characters observed from living materials (Table 2).

### 2.2. Phenetic Analyses

The phenetic analyses presented below summarize patterns of overall morphological similarity and differentiation based on Gower distances and should not be interpreted as estimates of phylogenetic relationships. The PCoA ordinations were derived from the same Gower distance matrices used for the corresponding UPGMA analyses. All analyses presented here are based exclusively on morphological data; no molecular sequence data were generated in the present study.

#### 2.2.1. Population-Level Analysis of Intraspecific Morphological Variation

**Figure 3:**
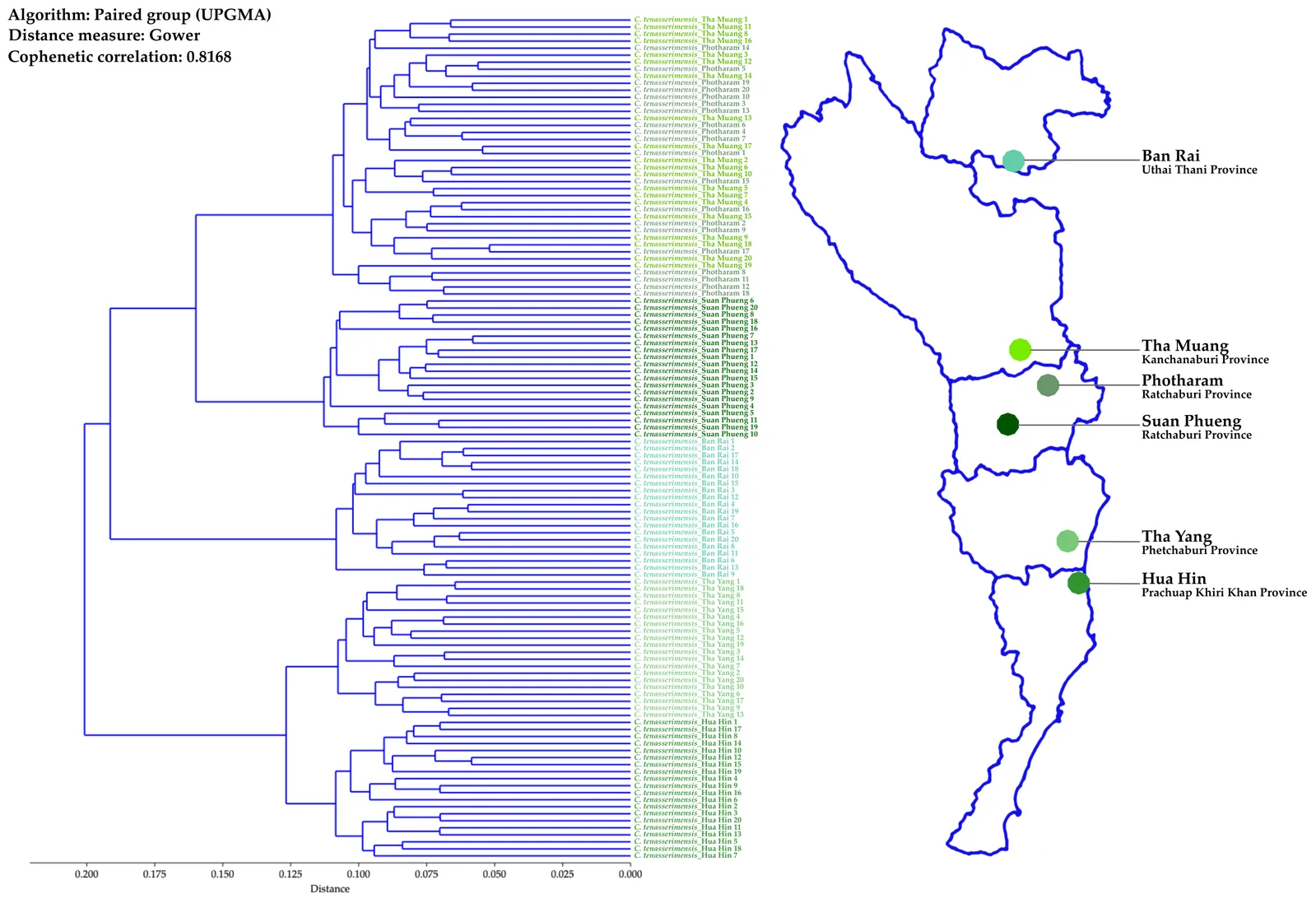
UPGMA dendrogram based on Gower distances showing morphological relationships among 120 individuals of *Curcuma tenasserimensis* from six populations: Suan Phueng, Tha Yang, Hua Hin, Tha Muang, Photharam, and Ban Rai (20 individuals per population). The accompanying map indicates the geographic locations of the sampled populations in western Thailand. The cophenetic correlation coefficient was 0.8168.

UPGMA clustering based on Gower distances revealed pronounced population-associated morphological structuring among the 120 individuals of *Curcuma tenasserimensis* sampled from six populations (Figure 3). Individuals from Suan Phueng, Ban Rai, Tha Yang, and Hua Hin showed relatively distinct clustering patterns, whereas those from Tha Muang and Photharam exhibited considerable intermixing within the same major cluster. The cophenetic correlation coefficient (*r* = 0.8168) indicated a good fit between the dendrogram and the original Gower distance matrix, as values greater than 0.8 indicate good correspondence between the dendrogram and the underlying distance matrix [18,19,20,21].

**Figure 4:**
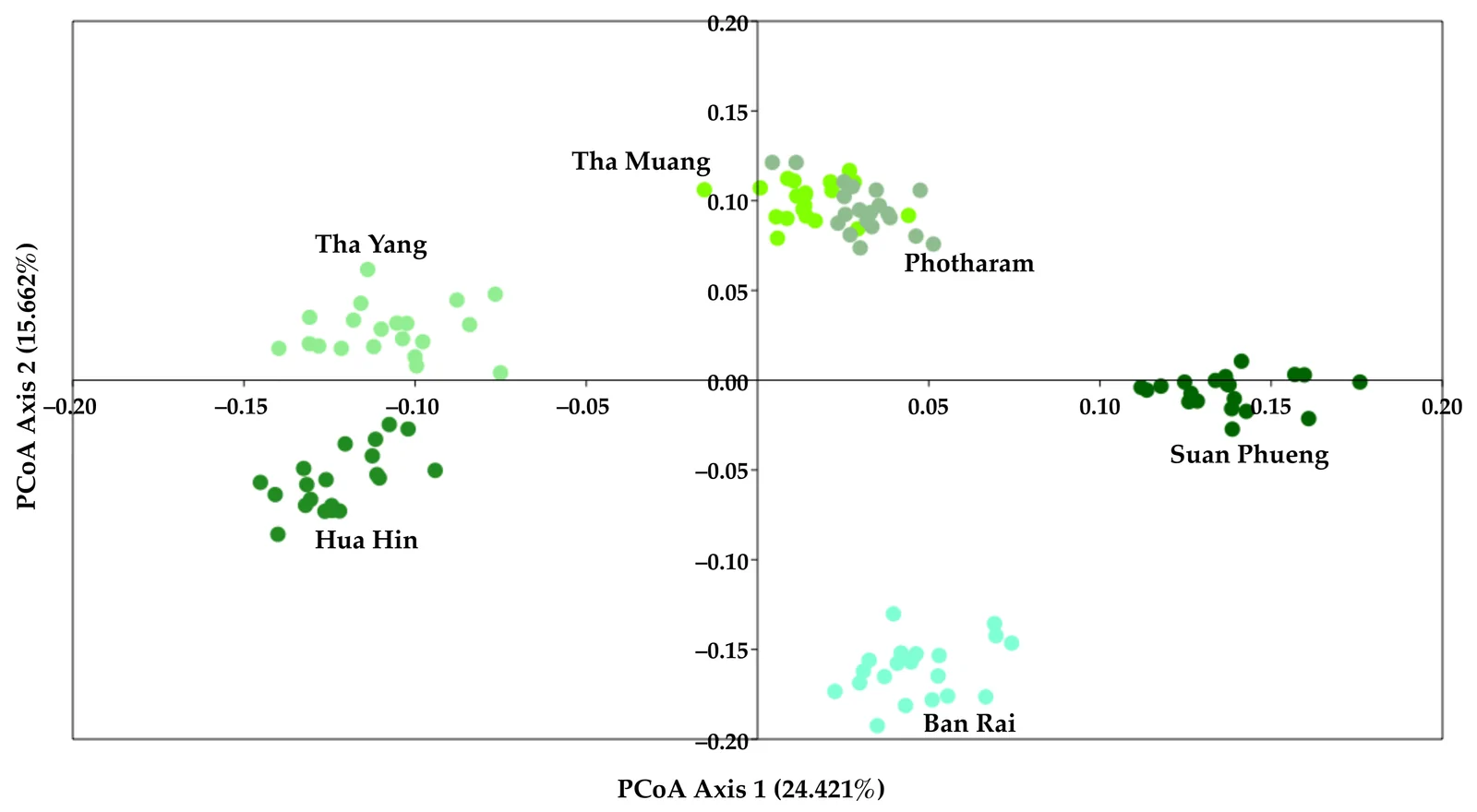
Principal coordinates analysis (PCoA) based on Gower distances showing morphological variation among 120 individuals of *Curcuma tenasserimensis* from six populations. Each population was represented by 20 individuals. PCoA Axis 1 and Axis 2 explained 24.421% and 15.662% of the total variation, respectively.

The PCoA provided a complementary ordination of individual-level morphological variation among the six populations (Figure 4). The first two axes accounted for 40.083% of the total variation, with Axis 1 and Axis 2 explaining 24.421% and 15.662%, respectively. Because the first two axes together represented only 40.083% of the total morphological variation, the two-dimensional ordination should be interpreted as a partial representation of the overall multivariate structure rather than as a complete summary of morphological differentiation. Suan Phueng, Ban Rai, Tha Yang, and Hua Hin occupied relatively distinct regions of the ordination space, whereas Tha Muang and Photharam showed substantial overlap. Thus, the ordination showed population-associated patterns of morphological variation, with particularly substantial overlap between Tha Muang and Photharam.

Examination of the underlying morphological data further showed that conspicuous among-population variation occurred in a subset of the 42 characters (Tables S1 and S2; Table A1). Among the qualitative characters, anthocyanin pigmentation of the fertile bracts (C29) varied between populations, being weak or restricted (state 1) in the Suan Phueng, Photharam, Tha Muang, and Tha Yang populations but absent (state 0) in Hua Hin and Ban Rai. Fertile-bract connation (C30) also varied geographically, with connation of approximately one-quarter to one-third of the bract length (state 1) in Suan Phueng, Photharam, and Tha Muang, whereas only basal to less than one-quarter connation (state 0) occurred in Tha Yang, Hua Hin, and Ban Rai. Among the quantitative characters, particularly conspicuous among-population variation occurred in thyrse length (C05), flower length (C08), calyx length (C09), floral tube length (C11), and lateral staminode length and width (C14–C15). In contrast, most remaining qualitative characters were invariant across the six populations. These patterns identify characters associated with the observed population-level morphological variation but do not represent character loadings on the PCoA axes.

#### 2.2.2. Individual-Level Analysis of Interspecific Morphological Differentiation

**Figure 5:**
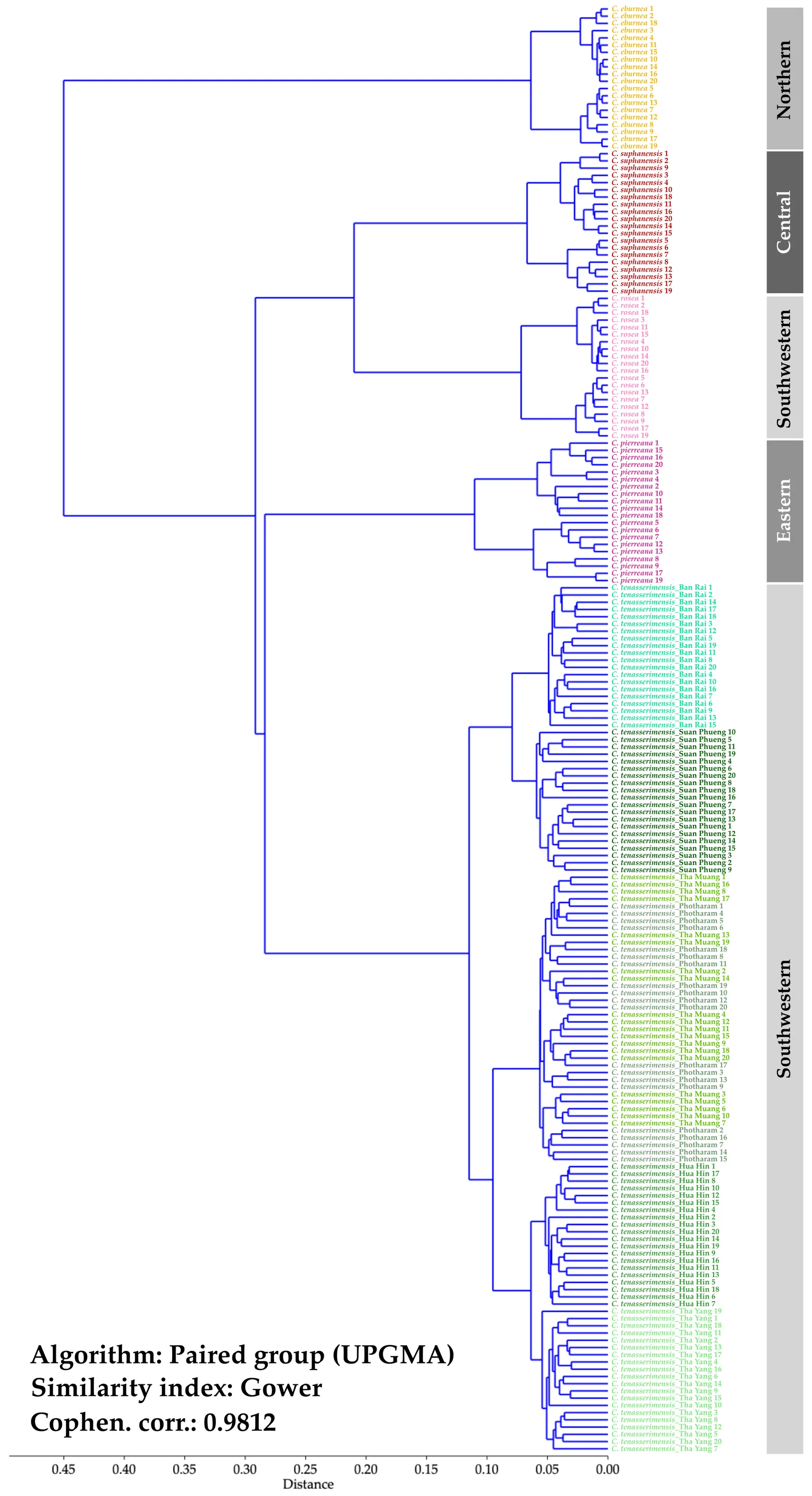
UPGMA dendrogram based on pairwise Gower distances among 200 individuals of *Curcuma tenasserimensis* and four morphologically allied species. The dataset comprised 120 individuals of *C. tenasserimensis* from six populations (20 individuals per population) and 20 individuals each of *C. pierreana*, *C. suphanensis*, *C. rosea*, and *C. eburnea*. Colors indicate taxa and populations. The cophenetic correlation coefficient was 0.9812.

The UPGMA analysis based on Gower distances revealed structured patterns of morphological similarity among the 200 individuals representing *Curcuma tenasserimensis* and four allied species (Figure 5). Individuals generally clustered according to their respective taxa. The 120 individuals of *C. tenasserimensis*, representing six populations, formed a cohesive major cluster, within which population-level substructure was evident. Individuals of *C. pierreana*, *C. suphanensis*, *C. rosea*, and *C. eburnea* each formed separate clusters. The cophenetic correlation coefficient (*r* = 0.9812) likewise indicated a good fit between the dendrogram and the original Gower distance matrix, exceeding the 0.8 threshold for good correspondence [19,21] (Figure 5).

**Figure 6:**
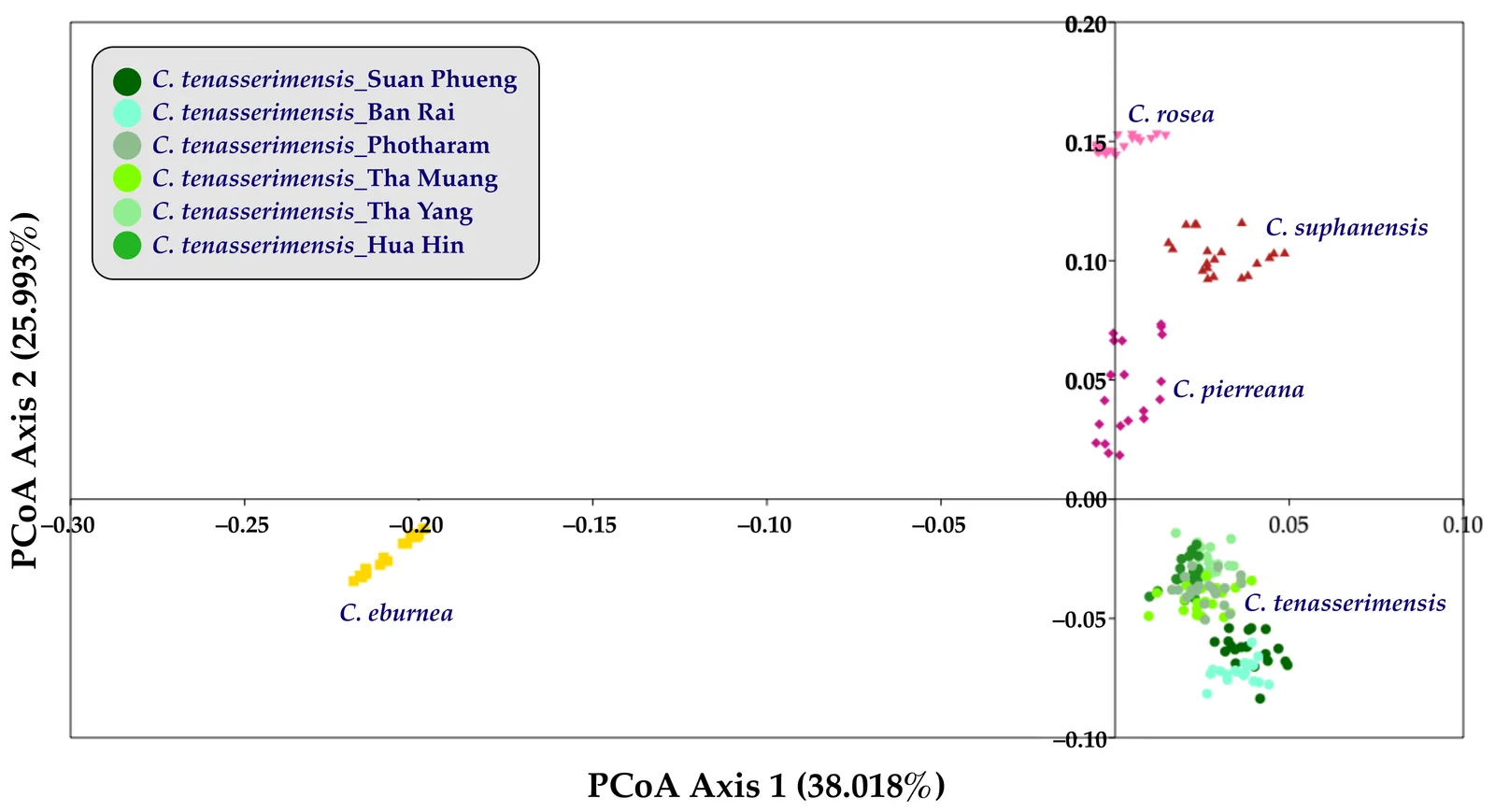
Principal coordinates analysis (PCoA) based on pairwise Gower distances among 200 individuals of *Curcuma tenasserimensis* and four morphologically allied species. PCo1 and PCo2 explained 38.018% and 25.993% of the total variation, respectively, accounting for 64.011% cumulatively. Colors and symbols indicate taxa and populations.

The PCoA showed a complementary pattern of morphological differentiation among the examined individuals (Figure 6). The first two principal coordinates accounted for 64.011% of the total variation, with PCo1 explaining 38.018% and PCo2 explaining 25.993%. Individuals of *C. tenasserimensis* occupied a relatively cohesive region of the ordination space, with partial overlap among the six populations. The four allied species occupied different regions of the ordination space. *Curcuma eburnea* was positioned primarily toward the negative side of PCo1, whereas *C. rosea*, *C. suphanensis*, and *C. pierreana* were positioned toward the positive side of PCo2 to varying degrees (Figure 6). Overall, the UPGMA and PCoA provide descriptive summaries of morphological similarity and differentiation among the examined individuals and taxa rather than formal statistical tests of species boundaries.

## 3. Discussion

### 3.1. Morphological Delimitation of Curcuma Tenasserimensis

Species delimitation in *Curcuma* subgen. *Ecomatae* can be challenging because morphologically similar species may share several vegetative and floral features, while individual quantitative characters may show considerable variation [1]. Consequently, species recognition within the subgenus is most appropriately based on combinations of multiple characters rather than on a single diagnostic feature [2,3,4,5]. The present study shows that *C. tenasserimensis* possesses a consistent combination of vegetative, inflorescence, and floral characters that distinguishes it from the morphologically comparable species included in this study.

Overall morphological resemblance alone should therefore be interpreted with caution when assessing species boundaries in subgen. *Ecomatae*. Several currently recognized species may appear superficially similar in general habit or floral appearance but remain distinguishable by consistent combinations of more detailed morphological characters. For example, *C. maxwellii* Škorničk. & Suksathan and *C. rubroaurantiaca* Škorničk. & Soonthornk. share a broadly similar general appearance yet are recognized as distinct species based on a combination of diagnostic morphological differences [22]. A comparable case is provided by *C. borealis* Saensouk, P.Saensouk & Boonma and *C. ecomata* Craib: the former had previously been confused with the latter because of their overall morphological resemblance, but detailed examination revealed consistent differences in multiple vegetative and reproductive characters, supporting their recognition as distinct species [4]. These examples illustrate that superficial resemblance or overlap in individual characters does not, by itself, constitute sufficient evidence for conspecificity.

Among these characters, the prominently plicate laminae, absence of coma bracts, pale fertile bracts, and spurred anthers with a thick rounded crest give *C. tenasserimensis* a superficial resemblance to *C. eburnea*. The inflorescence architecture provides an additional field-recognition character: in *C. tenasserimensis*, the peduncle remains largely enclosed by the leaf sheaths and the basal portion of the thyrse is further enclosed by sterile basal bracts, whereas in *C. eburnea* the peduncle and basal portion of the thyrse are clearly exserted from the leaf sheaths. Nevertheless, the two species differ consistently in several taxonomically informative reproductive characters. *Curcuma tenasserimensis* has fewer fertile bracts that are less extensively connate, a longer and puberulent calyx, an obovate and shortly hairy labellum with a distinctive yellow distal region fading proximally, a longer filament, longer epigynous glands, and a puberulous to pilose subglobose or oblate ovary. In contrast, *C. eburnea* has more numerous and more extensively connate fertile bracts, a shorter glabrous calyx, an obscurely trilobed glabrous labellum with a continuous bright yellow median band, a shorter filament and epigynous glands, and a cylindrical glabrous ovary. The presence of bracteoles in *C. tenasserimensis*, together with differences in the number of flowers per cincinnus, provides additional distinction between the two taxa. These differences involve several independent floral structures and therefore provide stronger evidence for taxonomic separation than differences based solely on dimensions or coloration.

Comparison with *Curcuma pierreana* is also taxonomically informative because the two species share several aspects of general habit and floral morphology. Nevertheless, they remain readily distinguishable by a consistent combination of characters. *Curcuma pierreana* [2] differs particularly in its shorter ligule, broadly ovate fertile bracts, reddish-purple coloration commonly developed toward the distal portions of the lateral staminodes and labellum, shorter filament, and differences in labellum marking. In *C. tenasserimensis*, the fertile bracts are ovate to lanceolate and cream, pale green, or pale pink, the lateral staminodes and labellum lack the characteristic distal reddish-purple coloration, the filament is approximately 5 mm long, and the labellum possesses a yellow distal portion that gradually fades proximally. These differences provide additional morphological characters for distinguishing the two species despite their overall resemblance.

### 3.2. Phenetic Evidence and Morphological Relationships

The individual-level interspecific analyses provide a quantitative summary of overall morphological similarity and differentiation among *Curcuma tenasserimensis* and the four morphologically allied species included for comparison. The analysis incorporated morphological variation directly from 200 measured individuals, comprising 120 individuals of *C. tenasserimensis* and 20 individuals each of *C. pierreana*, *C. suphanensis*, *C. rosea*, and *C. eburnea*. The UPGMA analysis grouped individuals largely according to their respective taxa, with the 120 individuals of *C. tenasserimensis* forming a cohesive major cluster while retaining population-level substructure. The four allied species formed separate clusters. Similarly, the PCoA placed individuals of *C. tenasserimensis* within a relatively cohesive region of the ordination space, whereas the four allied species occupied different regions. The first two PCoA axes accounted for 64.011% of the total morphological variation, indicating that the two-dimensional ordination summarized a substantial, although incomplete, proportion of the multivariate distance structure.

The broad agreement between the UPGMA and PCoA patterns shows that the observed morphological differentiation is evident in two complementary representations of the same Gower distance matrix. The cophenetic correlation coefficient of 0.9812 further indicates a close correspondence between the UPGMA dendrogram and the underlying pairwise Gower distances. Nevertheless, these results should be interpreted as descriptive summaries of overall morphological similarity rather than as formal tests of species boundaries or hypotheses of evolutionary relationships. Phenetic clustering groups operational taxonomic units according to observed similarity and does not, by itself, distinguish similarities resulting from common ancestry from those arising through convergence or parallelism [19,20,21]. Accordingly, the phenetic analyses complement the conventional taxonomic assessment by quantitatively summarizing patterns across the 42 morphological characters, but they do not replace character-based diagnosis or phylogenetic evidence [23].

### 3.3. Morphological Cohesion Among Geographically Separated Populations

An important aspect of the present study is that *C. tenasserimensis* is not known from a single locality. The six populations examined extend across Uthai Thani, Kanchanaburi, Ratchaburi, Phetchaburi, and Prachuap Khiri Khan provinces, covering a substantial portion of the Southwestern floristic region of Thailand [2,11]. Despite this geographical separation, the populations retain the principal morphological features used to characterize the species, although variation occurs in several quantitative characters and in the degree of pigmentation of some structures.

The individual-level analyses provide a direct assessment of morphological variation across all 120 measured individuals from the six populations. Although some population-level structuring was evident, substantial overlap and close morphological association occurred among several populations, and the individuals of *C. tenasserimensis* remained broadly differentiated from those of the comparative species in the interspecific analyses. This pattern is relevant to species delimitation because it suggests that the observed geographical variation among populations is smaller, in overall morphological terms, than the differentiation separating *C. tenasserimensis* from the recognized comparative species. The individual-level results therefore support the interpretation of the geographically separated populations as components of a single morphologically variable species rather than as several independently differentiated taxa.

Some degree of morphological variation among geographically separated populations is expected in widespread plant species and may reflect environmental conditions, phenotypic plasticity, local differentiation, or interactions among these factors [24,25]. In *Curcuma* and other seasonally dormant Zingiberaceae, variation in the dimensions of vegetative structures may also partly reflect differences in developmental condition and local environmental factors, as vegetative growth is responsive to temperature, water availability, and light regime [26,27]. This interpretation is also supported by the population-level descriptive statistics, which show substantial overlap in the observed ranges of many quantitative characters among populations (Table S2). Accordingly, moderate differences in lamina size, thyrse dimensions, flower dimensions, or pigmentation should be interpreted cautiously when they are not accompanied by consistent differentiation in structural characters. In *C. tenasserimensis*, the stability of the principal qualitative diagnostic features across populations is therefore taxonomically more informative than minor differences in individual measurements.

Taken together, the population-level and interspecific individual-level analyses provide complementary perspectives on morphological variation in *Curcuma tenasserimensis*. The population-level analysis reveals morphological variation and structuring among the sampled populations, whereas the interspecific analysis places this variation in the broader context of morphological differentiation from the four allied species examined. Although the six populations show measurable variation in several quantitative and qualitative characters, the principal diagnostic character combination remains consistent across populations, and the 120 individuals collectively form an overall morphologically cohesive assemblage in the interspecific analyses. These results are therefore consistent with the interpretation of the geographically separated populations as a single morphologically variable species.

### 3.4. Taxonomic and Biogeographic Significance

The confirmed distribution of *C. tenasserimensis* across five provinces indicates that the species is associated with a broader geographical area of Southwestern Thailand rather than representing a narrowly localized endemic known from a single site [2,8,9,13,14]. Its occurrence mainly in mixed evergreen forest and along forest margins on low hills at approximately 50–320 m a.s.l. suggests an ecological association with low-elevation forest habitats within this floristic region. The distribution of *C. tenasserimensis* also falls within the broader geographical context of the Tenasserim (Tanaosri) Range, a botanically distinctive mountainous region of western and southwestern Thailand. The importance of this region as a source of geographically distinctive plant diversity is reflected by several taxa whose names refer directly to the Tenasserim or Tanaosri landscape, such as *Artabotrys tanaosriensis* Jun H.Chen, Chalermglin & R.M.K.Saunders [28], *Kanburia tenasserimensis* (Mattapha & Sirich.) J.Compton, Mattapha, Sirich. & Schrire [29,30], *Scutellaria tenasserimensis* A.J.Paton [31], and *Sonerila tenasserimensis* J.Wai & J.M.Hu [32].

Continued discoveries of geographically restricted or previously overlooked taxa of *Curcuma* in Thailand and neighboring parts of mainland Southeast Asia indicate that diversity within subgen. *Ecomatae* remains incompletely documented [5,6,8,9,18]. The recognition of *C. tenasserimensis* further highlights the importance of geographically extensive field sampling. Had the taxon been evaluated from only a single population, it would have been more difficult to determine whether differences in quantitative morphology represented species-level characters or local variation. Examination of six populations instead provided a broader basis for distinguishing the stable diagnostic character combination from population-level variation.

The occasional occurrence of inflorescences of *C. tenasserimensis* in local markets in Kanchanaburi and Ratchaburi suggests that the species may also be locally utilized. Although the present study was not designed as an ethnobotanical investigation, this observation highlights the practical importance of accurate species identification for documenting plant utilization and provides a basis for future studies of its local uses and sustainable management. Recognition and documentation of the species may therefore contribute not only to taxonomic knowledge but also to the conservation and responsible utilization of regional plant resources.

The present study also illustrates the value of combining detailed taxonomic morphology with multivariate phenetic analyses for documenting patterns of morphological differentiation among closely similar taxa. Morphological evidence continues to play a central role in the discovery, delimitation, diagnosis, and description of new species, particularly in plants. Ziegler et al. [23] showed that most recently described plant species in their broad-scale survey were still delimited and described using morphological evidence alone, while species based exclusively on molecular evidence remained uncommon; their findings further suggest that many morphologically distinct species remain to be discovered. Beyond formal species description, morphology remains fundamental in practical plant identification because observable vegetative and reproductive characters provide the first and most immediately accessible basis for recognizing taxa in the field and herbarium. Accurate identification is important not only for taxonomic research but also for local communities that depend on plants for food, medicine, ornamentals, and other uses, where misidentification may lead to inappropriate or unsafe utilization [14,33,34]. Reliable species-level identification is likewise essential for governmental agencies, conservation organizations, and land managers responsible for biodiversity assessment, monitoring, management, and protection. Correctly recognized taxa provide the basic units for documenting distributions, evaluating conservation status, setting management priorities, and identifying habitats that require protection; thus, the value of sound morphological taxonomy extends beyond the species themselves to the conservation and management of the natural habitats in which they occur [35,36]. In this context, the formal recognition and reliable identification of *Curcuma tenasserimensis* provide an important foundation for future ecological monitoring, assessment of local uses, and conservation planning across its known range in Southwestern Thailand. Quantitative phenetic analyses can therefore provide a complementary framework for evaluating overall morphological differentiation among putative new taxa and morphologically similar recognized species.

### 3.5. Study Limitations and Future Research

The present study has several limitations that should be considered when interpreting the results. First, the taxonomic recognition of *Curcuma tenasserimensis* was based on comparative morphology complemented by multivariate phenetic analyses, without molecular sequence data. Although the morphological evidence supports the distinction of *Curcuma tenasserimensis* from the comparative taxa included in this study, molecular phylogenetic analyses would provide an important additional line of evidence for evaluating its evolutionary relationships within subgen. *Ecomatae*.

Second, the UPGMA and PCoA analyses were intended as descriptive summaries of overall morphological similarity and differentiation rather than as tests of species boundaries or phylogenetic relationships. In particular, the UPGMA dendrogram represents hierarchical clustering based on overall morphological similarity and should not be interpreted as a phylogenetic tree or as evidence of common ancestry among the included taxa. Likewise, the PCoA ordinations provide graphical representations of the underlying morphological distance structure rather than evolutionary relationships. The first two PCoA axes represent only part of the total morphological variation, and character-level associations with these axes were not evaluated. Consequently, the relative contributions of individual morphological characters to PCoA Axes 1 and 2 cannot be determined from the present ordinations. Although the underlying data and population-level summary statistics identify several characters showing conspicuous among-population variation, these patterns should not be interpreted as character loadings on the PCoA axes.

Furthermore, although six geographically separated populations and 120 individuals of *C. tenasserimensis* were examined, morphological measurements were obtained from plants under natural field conditions. The present dataset therefore cannot fully distinguish genetically based morphological differentiation from environmentally induced phenotypic plasticity. Common-garden cultivation experiments, ideally combined with population-level molecular analyses, would help clarify the relative contributions of genetic and environmental factors to the observed morphological variation.

Long-term monitoring of the six known populations is also needed to evaluate population trends, habitat changes, and potential threats more robustly. Finally, ethnobotanical surveys of communities within the species’ known range would be valuable for determining whether *C. tenasserimensis* is locally recognized or utilized and for assessing whether harvesting may represent a potential conservation concern.

## 4. Materials and Methods

### 4.1. Field Surveys and Plant Material

**Figure 7:**
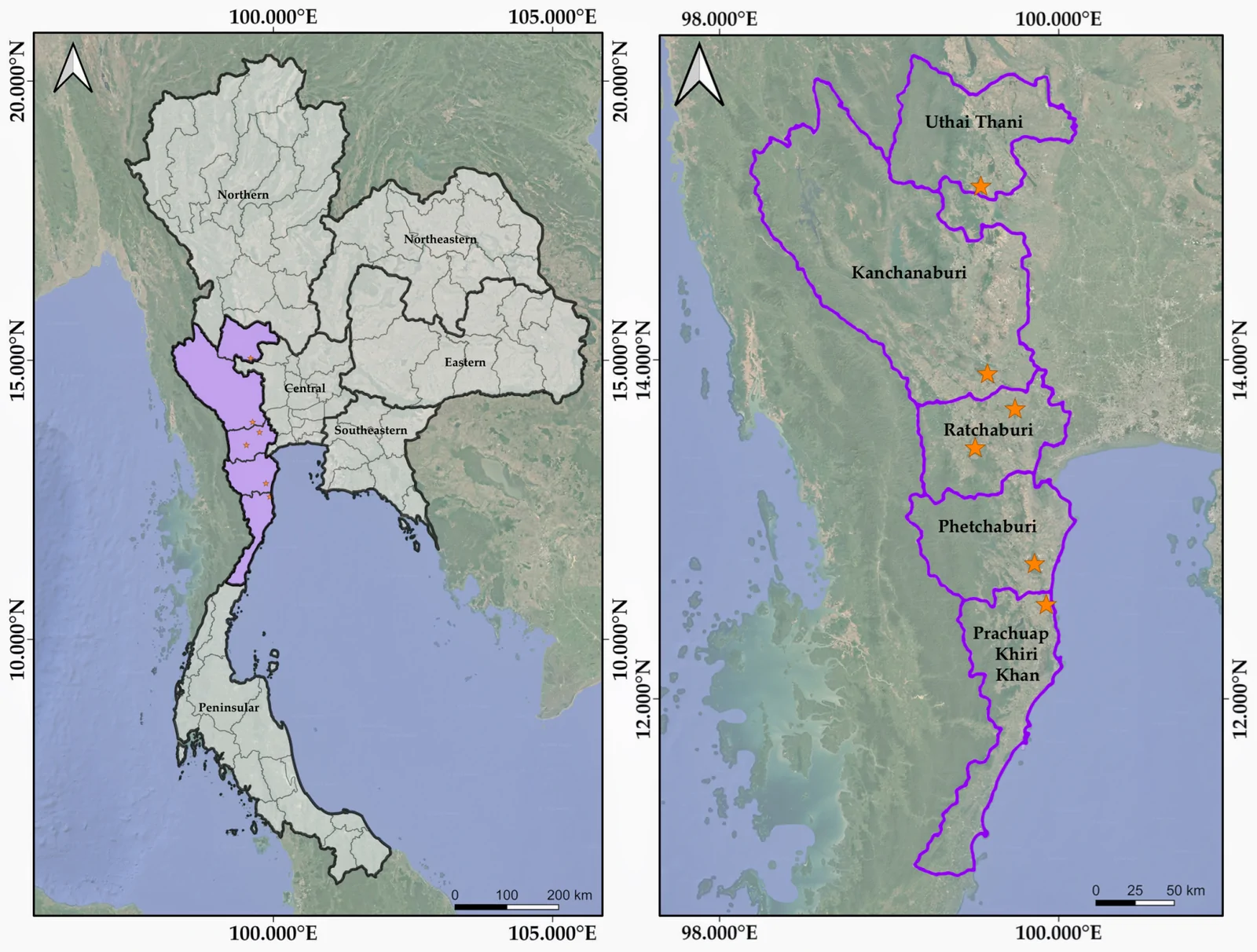
Distribution of *Curcuma tenasserimensis* Boonma, Saensouk & P.Saensouk, sp. nov., in Southwestern Thailand. The left panel shows the Southwestern floristic region of Thailand (purple shading) within the country, while the right panel shows the surveyed provinces within the region; orange stars indicate the six populations of *C. tenasserimensis* included in this study. Map created using QGIS version 3.34 with the WGS 84 geographic coordinate reference system (EPSG:4326). Map prepared by Kamonwan Koompoot.

Botanical surveys were conducted from 2021 to 2025 across the Southwestern floristic region of Thailand, including Uthai Thani, Kanchanaburi, Ratchaburi, Phetchaburi, and Prachuap Khiri Khan provinces (Figure 7).

Six geographically separated populations attributable to the putative new taxon were documented in Uthai Thani, Kanchanaburi, Ratchaburi, Phetchaburi, and Prachuap Khiri Khan provinces. Other *Curcuma* populations encountered during the surveys were identified separately and were not included in the population-level assessment of the putative new taxon. The populations attributable to the putative new taxon were found mainly in mixed evergreen forest and along forest margins on low hills at elevations of approximately 50–320 m a.s.l. Field observations were conducted during flowering and fruiting periods to document vegetative and reproductive morphology, phenology, habitat characteristics, and observable threats. For the purposes of this study, a mature individual was defined as a plant bearing an inflorescence with at least one flower at anthesis. At each of the six populations, 20 such mature individuals were examined and measured directly in the field, resulting in a total sample of 120 individuals (*n* = 20 per population). These individuals constituted the complete individual-level dataset used to assess within- and among-population morphological variation. Five of these 20 measured individuals per population were selected as representative collections: two were prepared as dried herbarium specimens, two were preserved in spirit, and all four preserved specimens were deposited at VMSU, while one individual was maintained as a living accession. Living plants were cultivated at Brio Botanical Research Garden, Ban Na, Nakhon Nayok Province, Thailand. Voucher specimens were prepared following standard herbarium procedures [37] and deposited at the Vascular Plant Herbarium, Mahasarakham University (VMSU), Thailand.

The limited removal of material for voucher preparation and living collections was not considered to impose measurable disturbance on the surveyed populations. Each population was estimated in the field to contain more than 200 mature individuals, and reproductive activity was evident from the presence of fruiting individuals, seedlings or young plants, and vegetative recruitment through lateral shoots. Only a small proportion of the observed population was therefore removed for scientific documentation, and no evidence of large-scale removal from the wild was observed during the surveys.

Among the examined populations, material from Suan Phueng District, Ratchaburi Province, was designated as the type collection because it comprised well-developed individuals with complete vegetative and reproductive structures suitable for taxonomic description and nomenclatural typification. The holotype was deposited at VMSU, with duplicate material preserved as indicated in the taxonomic treatment.

Geographic coordinates and elevations were recorded during field surveys. Precise geographic coordinates are withheld from the published taxonomic treatment to reduce potential collection pressure on the known wild population. The complete coordinates of the type locality are permanently retained in the authors’ original field records and on the label of the holotype specimen deposited at the Vascular Plant Herbarium, Mahasarakham University (VMSU).

### 4.2. Morphological Observations and Measurements

Morphological observations were based primarily on mature living plants examined in the field and were supplemented by freshly collected flowering and fruiting material, spirit-preserved flowers, dried herbarium specimens, and ex situ cultivated plants. Morphological measurements were taken using a ruler and vernier caliper (Mitutoyo Corporation, Kawasaki, Japan), as appropriate. Floral structures were dissected and examined under a stereomicroscope (Stemi 2000-C; Zeiss, Oberkochen, Germany). Measurements of floral structures were taken from freshly opened flowers whenever possible to minimize changes in size, shape, colour, and posture resulting from senescence or preservation.

The characters examined included habit, rhizome and rhizome branches, roots and root tubers, leafy shoot, bladeless sheaths, leaf sheaths, ligule, petiole, lamina, inflorescence, peduncle, thyrse, fertile bracts, cincinni, flowers, calyx, floral tube, corolla lobes, lateral staminodes, labellum, filament, anther, anther crest, anther spurs, epigynous glands, stigma, ovary, fruit, and seeds. Colors were recorded from fresh material under natural daylight because some colors may change after preservation or drying. Morphological terminology generally follows Beentje [38].

Morphological variation was initially evaluated separately for each of the six populations. Characters consistently expressed among geographically separated populations were considered in assessing morphological cohesion, whereas quantitative variation was incorporated into the overall range presented in the species description.

### 4.3. Herbarium Study and Comparative Taxonomic Sources

Comparative taxonomic assessment was conducted using herbarium specimens, type material, protologues, original descriptions, subsequent taxonomic treatments, living collections, field observations, and high-resolution digital specimen images. Material from the following herbaria and institutional collections was consulted either directly or through digital resources: A, AAU, B, BK, BKF, BM, BR, C, CAL, DMSC, E, HITBC, HN, HNPM, HNU, IBK, IMDY, K, KKU, L, NY, P, QBG, RAF, SGN, SING, TNS, US, VMSU, VNM, and VNMN. Herbarium acronyms follow Index Herbariorum [39].

The newly collected material was compared with recognized species of *Curcuma* subgen. *Ecomatae*, with particular attention to morphologically similar taxa. Detailed conventional taxonomic comparison focused especially on *C. pierreana* Gagnep. [2] and *C. eburnea* Škorničk., Suksathan & Soonthornk. [40]. For the individual-level interspecific phenetic analysis, four morphologically allied species, *C. pierreana*, *C. suphanensis*, *C. rosea*, and *C. eburnea*, were selected to provide a comparative framework for evaluating the morphological differentiation of *C. tenasserimensis*. Species identities and character states were verified against protologues and the subsequent taxonomic literature wherever available [2,4,5,7,40,41].

### 4.4. Morphological Dataset and Character Coding

A morphological dataset comprising 42 characters was assembled for quantitative assessment of the putative new taxon and selected morphologically comparable members of *Curcuma* subgen. *Ecomatae*. The dataset comprised 22 quantitative and 20 qualitative characters. The qualitative characters consisted of five binary, three ordinal, and 12 nominal variables. Characters were selected from vegetative, inflorescence, and floral morphology and were chosen to capture variation considered informative for distinguishing the included taxa.

Characters showing no informative variation among the included taxa, those that could not be scored reliably across all taxa, and those considered strongly dependent on developmental stage or preservation were excluded from the final analysis. Bracteole characters were not included in the phenetic dataset because they could not be scored consistently across all individuals and taxa included in the interspecific analysis. Although bracteoles could be directly examined and measured in *C. tenasserimensis* and in some comparative material, equivalent observations were not consistently available across the complete comparative dataset. Their inclusion would therefore have introduced substantial missing or non-comparable data into the individual-level matrix. Accordingly, the phenetic analyses were restricted to characters that could be scored consistently across the comparative dataset, whereas bracteole morphology was retained for conventional taxonomic comparison and identification, where it remains diagnostically informative. Qualitative character states were coded numerically solely for analytical purposes and were not intended to imply evolutionary polarity or transformation sequences.

For the individual-level datasets, quantitative characters were represented by measurements obtained directly from each individual, whereas qualitative characters were scored according to the states observed in each individual. The same 42-character framework, character definitions, and coding criteria were applied consistently to both the intraspecific and interspecific analyses. For the four comparative species, individual-level morphological data were obtained from 20 measured individuals per species using the same character definitions and scoring criteria applied to *Curcuma tenasserimensis*, thereby maintaining consistency across the complete comparative dataset.

### 4.5. Phenetic Study

Morphological dissimilarity among OTUs was quantified using the Gower coefficient [19], which was selected because the dataset comprised a mixture of quantitative, binary, ordinal, and nominal morphological variables. For each pair of OTUs, character-wise dissimilarities were calculated according to variable type and combined across the characters available for comparison, with quantitative variables standardized by their observed ranges so that characters measured on different scales could contribute comparably to the overall distance. The resulting Gower distances range from 0 to 1, with values approaching 0 indicating greater overall morphological similarity and values approaching 1 indicating greater morphological dissimilarity. The resulting distance matrix was subjected to hierarchical clustering using the unweighted pair-group method with arithmetic mean (UPGMA) [20] and to principal coordinates analysis (PCoA). Phenetic analyses were performed using PAST version 4.15 [42].

Two complementary individual-level phenetic analyses were conducted. First, an intraspecific analysis was performed using 120 measured individuals of *Curcuma tenasserimensis* from six geographically separated populations (20 individuals per population). Each individual was treated as an operational taxonomic unit (OTU). Quantitative characters were represented by measurements obtained directly from each individual, whereas qualitative characters were scored individually according to the observed character states. This analysis was used to evaluate patterns of morphological variation and cohesion among populations of *C. tenasserimensis*.

Second, an interspecific individual-level analysis was conducted using 200 measured individuals, comprising the same 120 individuals of *C. tenasserimensis* and 20 individuals each of four morphologically allied species, *C. pierreana*, *C. suphanensis*, *C. rosea*, and *C. eburnea*. Each individual was treated as an OTU using the same 42-character framework. UPGMA and PCoA were used descriptively to summarize patterns of overall morphological similarity and differentiation among individuals and taxa.

The complete individual-level morphological dataset used in the intraspecific analysis is provided in Supplementary Table S1, with population-level summary statistics for the quantitative characters provided in Supplementary Table S2 and the corresponding pairwise Gower distance matrix in Supplementary Table S3. The complete individual-level morphological dataset used in the interspecific analysis is provided in Supplementary Table S4, with the corresponding pairwise Gower distance matrix provided in Supplementary Table S5.

All multivariate analyses were interpreted strictly as measures of overall morphological similarity and differentiation. The resulting clusters and ordination patterns were not interpreted as estimates of phylogenetic relationships.

### 4.6. Distribution Mapping

The distribution map was prepared using QGIS version 3.34 with the WGS 84 geographic coordinate reference system (EPSG:4326). Confirmed distribution records of *C. tenasserimensis* were based exclusively on field observations and voucher-supported collections from Uthai Thani, Kanchanaburi, Ratchaburi, Phetchaburi, and Prachuap Khiri Khan provinces. Only records verified from examined material were included in the mapped distribution. Precise coordinates were not displayed to minimize potential collection pressure on wild populations.

### 4.7. Preliminary Conservation Assessment

The preliminary conservation status of *C. tenasserimensis* was evaluated following the IUCN Red List Categories and Criteria and the current Guidelines for Using the IUCN Red List Categories and Criteria [15]. The assessment considered the number and geographical distribution of confirmed populations, field observations of population abundance, habitat condition, observed or potential anthropogenic threats, and the availability of potentially suitable habitat. Population information was derived from repeated field observations conducted between 2021 and 2025 rather than from a complete demographic census. The proposed conservation status is therefore regarded as preliminary and may require reassessment when additional distributional and population data become available.

## 5. Conclusions

This study describes *Curcuma tenasserimensis* as a new species of *Curcuma* subgen. *Ecomatae* from Southwestern Thailand based on detailed comparative morphology complemented by multivariate phenetic analyses. The species is distinguished from morphologically allied taxa by a consistent combination of vegetative, inflorescence, floral, and reproductive characters. Individual-level phenetic analyses based on 42 morphological characters provided complementary perspectives on both intraspecific variation and interspecific differentiation. Analysis of 120 individuals from six geographically separated populations revealed population-associated morphological variation while retaining overall morphological cohesion within *C. tenasserimensis*. In the interspecific analysis of 200 individuals, comprising the same 120 individuals of *C. tenasserimensis* and 20 individuals each of four morphologically allied species, individuals of *C. tenasserimensis* formed an overall cohesive morphological assemblage differentiated from the comparative species. Together, the conventional taxonomic comparison and quantitative phenetic analyses are consistent with the recognition of *C. tenasserimensis* as a morphologically distinct species.

The discovery of *C. tenasserimensis* further contributes to the documented diversity of subgen. *Ecomatae* and highlights the botanical significance of the Tenasserim (Tanaosri) landscape of Southwestern Thailand. Its recognition provides a taxonomic foundation for reliable identification, future ecological and population studies, assessment of potential local uses, and conservation planning for both the species and its natural habitats. The documented occurrence of inflorescences in local markets further emphasizes the relevance of accurate taxonomic identification to the documentation, conservation, and sustainable utilization of regional plant resources.

## Figures and Tables

**Table 1:** Morphological comparison of *Curcuma tenasserimensis* and the morphologically similar *C. eburnea* and *C. pierreana*.

Character	*C. tenasserimensis*	*C. eburnea*	*C. pierreana*
Rhizome internal color	Ivory	Pale yellow	Cream to pale yellow
Leafy shoot	30–50 cm tall	60–75 cm tall	20–40 cm tall
Ligule	6–8 mm	To 8 mm	c. 2 mm
Inflorescence emergence and basal enclosure	Mostly enclosed by leaf sheaths; basal part of thyrse additionally enclosed by sterile basal bracts	Exserted from leaf sheaths; basal part of thyrse exposed, not enclosed by leaf sheaths	Exserted from leaf sheaths; basal part of thyrse exposed, not enclosed by leaf sheaths
Fertile bracts	12–18, ovate to lanceolate	30–40, broadly ovate to trullate	8–20, broadly ovate
Fertile bract indumentum	Puberulent	Glabrous	Puberulent
Fertile bract connation	Lower 1/5–1/3	Lower 1/3–1/2	Lower 1/4–1/3
Flower length	4.5–6.1 cm	5–5.5 cm	5–6 cm
Calyx	15–20 mm Puberulent	8–10 mm Glabrous	c. 16 mm Scattered fine hairs
Floral tube	30–43 mm Scattered fine hairs	c. 35 mm Glabrous	32–37 mm Sparsely puberulent
Lateral staminodes	Ovate to broadly elliptic, 15–20 × 10–16 mm, ivory to cream	Irregularly ovate to rhomboid, c. 15 × 12 mm, white or yellowish centrally/apically	Broadly ovate to broadly elliptic, 15–17 × 12–14 mm, usually reddish purple apically
Labellum	Ivory to cream with a yellow distal part gradually fading towards approximately half of the median band, with short hairs denser along the margins of the median band	White with bright yellow median band, glabrous	White with bright yellow median band, usually with red-purple apically, glandular-hairy
Filament length	c. 5 mm	c. 2 mm	2–3 mm
Anther spurs length	c. 1 mm	1–1.5 mm	c. 1 mm
Epigynous glands length	c. 5 mm	c. 3 mm	c. 5 mm
Ovary	Subglobose to oblate, Puberulous to pilose	Cylindrical Glabrous	Globose to subglobose Finely hairy

**Table 2:** Key to *Curcuma tenasserimensis* and morphologically similar species of *Curcuma* subgen. *Ecomatae*.

Characters	Species
1a. Inflorescence lateral, arising separately from the leafy shoot	2
1b. Inflorescence central or terminal, associated with the leafy shoot	3
2a. Anther spurs absent	*C. candida*
2b. Anther spurs present	*C. singularis*
3a. Adaxial surface of lamina pubescent or puberulent	4
3b. Adaxial surface of lamina glabrous	6
4a. Fertile bracts pale purple to purple;	*C. pitukii*
4b. Fertile bracts green to whitish;	5
5a. Labellum with two pale purple patches; anther spurs c. 1 mm long, folding or curving backwards beneath the anther base	*C. retrocalcaria*
5b. Labellum without purple patches; anther spurs c. 0.5 mm long, directed downwards	*C. chantaranothaii*
6a. Fertile bracts glabrous	7
6b. Fertile bracts puberulent or otherwise hairy	8
7a. Ovary cylindrical and glabrous; labellum obscurely trilobed, with a continuous bright yellow median band	*C. eburnea*
7b. Ovary subglobose and pubescent; labellum obovate, basally richly tinged dark red to reddish orange	*C. peramoena*
8a. Lateral staminodes and labellum with reddish purple distally	*C. pierreana*
8b. Lateral staminodes and labellum without reddish purple distally	9
9a. Lamina usually with dark reddish midrib; bracteole absent	*C. rosea*
9b. Lamina usually with green midrib, rarely with reddish midrib; bracteole present	10
10a. Fertile bracts white with dark reddish distally; flowers slightly exserted from the bracts, 4.0–4.1 cm long; calyx c. 13 mm long; labellum ovate; filament 2.5–3 mm long	*C. suphanensis*
10b. Fertile bracts cream, pale green, or pale pink, without dark reddish distally; flowers well exserted from the bracts, 4.5–6.1 cm long; calyx 15–20 mm long; labellum obovate; filament c. 5 mm long	*C. tenasserimensis*

## Data Availability

The complete individual-level morphological datasets used in the intraspecific and interspecific phenetic analyses, population-level descriptive statistics, and the corresponding pairwise Gower distance matrices are provided in the Supplementary Materials. Morphological character definitions and coding criteria are provided in Table A1. Voucher specimens supporting the taxonomic treatment are deposited at VMSU, and living accessions are maintained at Brio Botanical Research Garden, Nakhon Nayok Province, Thailand. No molecular sequence data were generated in this study.

## Supplementary Materials

The following supporting information can be downloaded at: https://www.mdpi.com/article/10.3390/plants15193072/s1, Table S1: Individual-level morphological dataset for 120 individuals of *Curcuma tenasserimensis* from six populations used in the intraspecific phenetic analysis; Table S2: Population-level summary statistics for quantitative morphological characters of *C. tenasserimensis* based on 20 individuals per population, presented as mean ± standard deviation (minimum–maximum); Table S3: Pairwise Gower distance matrix derived from the individual-level morphological dataset of 120 individuals of *C. tenasserimensis*; Table S4: Individual-level morphological dataset for the interspecific phenetic analysis of 200 measured individuals, comprising 120 individuals of *C. tenasserimensis* and 20 individuals each of *C. pierreana*, *C. suphanensis*, *C. rosea*, and *C. eburnea*; Table S5: Pairwise Gower distance matrix derived from the individual-level interspecific dataset of 200 individuals.

## Abbreviations

The following abbreviations are used in this manuscript:

AOO	Area of occupancy
EOO	Extent of occurrence
OTU	Operational taxonomic unit
PCoA	Principal coordinates analysis
UPGMA	Unweighted pair-group method with arithmetic mean

## Appendix A

The morphological characters used in the phenetic analyses are defined in Table A1. The dataset includes quantitative and qualitative characters representing vegetative, inflorescence, and floral morphology, with qualitative character states numerically coded for analysis.

**Table A1:** Morphological characters, definitions, coding, and variable types used in the phenetic analyses of *Curcuma* subgen. *Ecomatae*.

Code	Character	Definition	Type	Unit	States/Coding
C01	Ligule length	Length of ligule.	Quantitative	mm	
C02	Lamina length	Length of mature lamina.	Quantitative	cm	
C03	Lamina width	Maximum width of mature lamina.	Quantitative	cm	
C04	Peduncle length	Length of peduncle.	Quantitative	cm	
C05	Thyrse length	Length of thyrse.	Quantitative	cm	
C06	Fertile bract length	Length of fertile bract.	Quantitative	cm	
C07	Fertile bract width	Maximum width of fertile bract.	Quantitative	cm	
C08	Flower length	Total flower length.	Quantitative	cm	
C09	Calyx length	Length of calyx.	Quantitative	mm	
C10	Calyx unilateral incision length	Length of unilateral calyx incision.	Quantitative	mm	
C11	Floral tube length	Length of floral tube.	Quantitative	mm	
C12	Dorsal corolla lobe length	Length of dorsal corolla lobe.	Quantitative	mm	
C13	Lateral corolla lobe length	Length of lateral corolla lobe.	Quantitative	mm	
C14	Lateral staminode length	Length of lateral staminode.	Quantitative	mm	
C15	Lateral staminode width	Maximum width of lateral staminode.	Quantitative	mm	
C16	Labellum length	Length of labellum.	Quantitative	mm	
C17	Labellum width	Maximum width of labellum.	Quantitative	mm	
C18	Filament length	Length of filament.	Quantitative	mm	
C19	Anther length	Length of anther as reported, including crest/spurs when stated.	Quantitative	mm	
C20	Epigynous gland length	Length of epigynous gland.	Quantitative	mm	
C21	Ovary length	Length of ovary.	Quantitative	mm	
C22	Rhizome branching habit	Overall architecture of rhizome branches.	Nominal		0 = compact/short; 1 = slender elongate branches; 2 = conspicuously creeping or numerous horizontal branches
C23	Adaxial lamina indumentum	Indumentum on adaxial lamina surface.	Nominal		0 = glabrous; 1 = hairy; 2 = variable
C24	Abaxial lamina indumentum	Indumentum on abaxial lamina surface.	Nominal		0 = glabrous; 1 = hairy; 2 = variable
C25	Ligule indumentum	Indumentum on ligule surface; marginal cilia alone treated as glabrous surface.	Nominal		0 = surface glabrous; 1 = surface hairy; 2 = variable
C26	Inflorescence position	Position of inflorescence relative to leafy shoot.	Binary		0 = central/terminal and associated with leafy shoot; 1 = lateral/separate from leafy shoot
C27	Fertile bract shape	Predominant fertile bract shape.	Nominal		0 = broadly ovate/trullate; 1 = ovate; 2 = lanceolate or narrowly ovate–lanceolate; 3 = triangular/ovate–triangular; 4 = other/variable
C28	Fertile bract indumentum	Presence of hairs on fertile bracts.	Binary		0 = glabrous; 1 = hairy
C29	Anthocyanin pigmentation of fertile bracts	Degree of pink/red/purple pigmentation of fertile bracts.	Ordinal		0 = absent; 1 = weak/restricted or variable tinge; 2 = conspicuous/predominant pink, red or purple
C30	Fertile bract connation	Approximate degree of connation at bract base.	Ordinal		0 = only at base to <1/4; 1 = ca. 1/4–1/3; 2 = >1/3–1/2
C31	Calyx indumentum	Indumentum of calyx.	Nominal		0 = glabrous; 1 = hairy; 2 = variable
C32	External floral tube indumentum	Presence of hairs on outer surface of floral tube.	Binary		0 = glabrous; 1 = hairy
C33	Lateral staminode shape	Predominant shape of lateral staminodes.	Nominal		0 = narrowly ovate/narrowly elliptic; 1 = ovate/broadly elliptic; 2 = rhomboid; 3 = obovate; 4 = irregular/asymmetrical/mixed
C34	Lateral staminode apex	Shape of lateral staminode apex.	Nominal		0 = acute; 1 = obtuse; 2 = rounded; 3 = variable/irregular
C35	Labellum shape	Overall outline of labellum.	Nominal		0 = ovate; 1 = obovate/broadly obovate; 2 = rectangular; 3 = obovate–rhomboid; 4 = mixed/other; 5 = obscurely trilobed
C36	Depth of labellum apical incision	Depth class of apical incision/sinus.	Ordinal		0 = retuse or ≤1 mm; 1 = >1–3 mm; 2 = >3–5 mm; 3 = >5 mm or incision ≥1/3 labellum length
C37	Yellow marking pattern of labellum	Configuration of principal yellow marking on labellum.	Nominal		0 = distal yellow area fading proximally; 1 = continuous median band; 2 = median band bifurcating/Y-shaped basally; 3 = median band plus additional patches/spots; 4 = central yellow area not reaching distal margin
C38	Ovary shape	Overall ovary shape.	Nominal		0 = subglobose/subspherical/oblate; 1 = cylindrical; 2 = ellipsoid/prolate ellipsoid; 3 = oblong/barrel-shaped
C39	Ovary indumentum	Presence of hairs on ovary.	Binary		0 = glabrous; 1 = hairy
C40	Anther-spur presence	Presence or absence of basal anther spurs.	Binary		0 = absent; 1 = present
C41	Anther-crest configuration	Overall configuration of anther crest.	Nominal		0 = obscure/reduced; 1 = rounded/obtuse; 2 = rectangular; 3 = retuse/bilobed; 4 = bicornuate; 5 = other
C42	Anther-spur length	Length of anther spur; 0 in spurless taxa.	Quantitative	mm	
